# Book Notices and Items

**Published:** 1861-03

**Authors:** 


Diphtheria. By Edward Headlam, Greenhow, London. Republished by Balliere Brothers, 440 Broadway, N. Y.

The above work, a neat little volume of 160 pages, was received from the publishers, but too late for notice in the February number of the *Journal.*

The author, who is a forcible writer, and seems well acquainted with the history, character and tendencies of epidemic diseases, traces the history of Diphtheria from the sixteenth century to the present time. Under this name, until recently, the disease was unknown; but in the symptoms of a disease known as "malignant sore throat, epidemic croup, and *morbus strangulatorius,*" given by writers in past generations, the author recognises the disease now known as *Diphtheria*, *Diphtheritis*, or *Diphtheritic Sore Throat.*

In the work before us, the disease is considered in regard to its sporadic and epidemic forms, its non-identity with scarlet fever, its coincidence with other human and brutal diseases, its communicability, &c., &c. The symptoms and "description of the several grades and varieties" are carefully given, with the appropriate treatment in each. In the close of the volume two chapters are devoted to the consideration of the sequelæ, and morbid anatomy.

We advise all who wish to study this formidable disease, in all its bearings, to forward Bailliere Brothers $1.25, the price of the work, for a copy.

---

☞ We are in receipt of "A Report on Epidemics and Endemics, read before the Medical Society of South-western New York, at its several sessions during 1848. By O. C. Gibbs, M.D., of Freesburg, N. Y." This is a pamphlet of 24 pages, well written, and giving many facts connected with the ravages of Plague and Yellow Fever. These, particularly the latter, are thought, by the author, to depend, for their production, on isothermal and terrene relations, and not contagion. We read the pamphlet with interest.

---

☞ The Committee from the Medical Society of Paris, appointed to investigate the subject of catheterism of the larynx in diphtheria, report against it. One of the subjects upon whom it was practiced, died from the operation. M. Loisean had previously recommended it.

---

☞ It is said that all ointments used for the cure of psora, owe their curative properties entirely to the lard. If this be true, the combinations of sulphur, mercury, &c., which are so often objected to, may be dispensed with.

